# Development of a data dictionary on viral hepatitis for the Philippines

**DOI:** 10.1093/oodh/oqae022

**Published:** 2024-07-17

**Authors:** Jan Michael M Herber, Randy Joseph G Fernandez, Geohari L Hamoy, Jhunnel P Macalanda, Gerard Paolo F Villanueva, Alvin B Marcelo, Janus P Ong

**Affiliations:** UPM Standards and Interoperability Lab (UPM SILab), National Institutes of Health, University of the Philippines Manila, 547 Pedro Gil Street, Ermita, Manila 1000, National Capital Region, Philippines; Department of Science and Technology, Philippine Science High School-Central Luzon Campus, Senator Miriam Defensor-Santiago Avenue (formerly Agham Road), Diliman, Quezon City 1104, National Capital Region, Philippines; UPM Standards and Interoperability Lab (UPM SILab), National Institutes of Health, University of the Philippines Manila, 547 Pedro Gil Street, Ermita, Manila 1000, National Capital Region, Philippines; UPM Standards and Interoperability Lab (UPM SILab), National Institutes of Health, University of the Philippines Manila, 547 Pedro Gil Street, Ermita, Manila 1000, National Capital Region, Philippines; UPM Standards and Interoperability Lab (UPM SILab), National Institutes of Health, University of the Philippines Manila, 547 Pedro Gil Street, Ermita, Manila 1000, National Capital Region, Philippines; UPM Standards and Interoperability Lab (UPM SILab), National Institutes of Health, University of the Philippines Manila, 547 Pedro Gil Street, Ermita, Manila 1000, National Capital Region, Philippines; UPM Standards and Interoperability Lab (UPM SILab), National Institutes of Health, University of the Philippines Manila, 547 Pedro Gil Street, Ermita, Manila 1000, National Capital Region, Philippines

**Keywords:** data dictionary, viral hepatitis, disease surveillance, terminology standards, health informatics, medical informatics, electronic medical records, interoperability, Philippines

## Abstract

The considerable difference between the reported number of actual and estimated viral hepatitis cases in the Philippines can be attributed to the lack of reliable data. Since viral hepatitis is endemic in the country, efficient disease control depends on access to quality data. The rapid adoption of electronic medical records in different facilities in the country offers opportunities for access to such data but raises concerns about its standardization for analysis. This study presents the steps taken toward the adoption of a standardized data dictionary for viral hepatitis, referencing existing international terminology standards. A series of workshops were conducted to gain consensus among subject matter experts such as epidemiologists, hepatologists and informaticists. These workshops provided context to the different stakeholders through a shared understanding of viral hepatitis care processes and transforming these into computable representations. Using Department of Health-approved paper forms for viral hepatitis surveillance, a multidisciplinary team mapped the 125 unique data elements to various international standard code sets. The output was a draft Philippine Viral Hepatitis Data Dictionary—a prerequisite for the semantic interoperability of data from different electronic medical records.

## INTRODUCTION

Viral hepatitis is endemic in the Philippines. This disease affected 0.97 in 100 000 Filipinos in 2019, although several regions have much higher infection rates: 4.65 in Soccsksargen, 4.89 in the Zamboanga Peninsula and 6.23 in Caraga [1]. Widespread testing in 2020, however, indicated that the reactivity rate for hepatitis B was 3.51% (out of 1 148 331 tests), while for hepatitis C it was 0.94% (out of 219 486 tests) [2]. On the other hand, recent estimates show that 9.8% of Filipinos (~10.143 million) have chronic hepatitis B [3], and an additional 0.4% (~439 000) have chronic hepatitis C [4]. The glaring difference between collected and estimated data exposes issues with the reliability of available data. On the other hand, even with the existence of available data, variances can also come from various factors such as recency, representativeness of included data, different modeling assumptions and the overtime change of clinical definitions [5]. In addition, collected and estimated data would differ in the correlation of population parameters (e.g. demographics vs. incidence), in part due to modeling assumptions in the estimated data. The World Health Organization (WHO) framework for global action underscores the importance of available data, noting the lack of adequate surveillance systems for evidence-based policy decisions [6].

Information systems such as electronic medical records (EMRs) allow clinicians to store data in digital format. Combined with prompt alert systems, EMRs improve screening rates for viral hepatitis [7]. This strengthens surveillance toward eventual disease control. However, the rapid increase in the adoption of EMRs raises concerns about the quality of data for analysis. Data quality can be assessed through the following data quality domains: accuracy, completeness, consistency, credibility and timeliness [8]. Data consistency, one of the specified domains, is difficult to achieve due to different EMR vendors among health facilities. These data consistency issues can be addressed by creating a shared repository that explains what the data mean and their relationship with other data—a data dictionary [9].

Creating a data dictionary involves adopting existing terminology standards and aligning other terminologies from existing information systems [10]. A consensus among subject matter experts (e.g. epidemiologists and hepatologists for viral hepatitis) ensures proper guidance in developing these health data standards.

## MATERIALS AND METHODS

A desk review of relevant policies of the Department of Health (DOH) and the Philippine Health Insurance Corporation (PhilHealth) was performed. A draft data dictionary and other machine-readable representations were developed from these policies, which were, in turn, vetted through consensus by a group of experts.

Two key policies were used for the development of viral hepatitis data dictionaries.

1. DOH Memorandum No. 2021-0381. Interim Guidelines on the Use of the One HIV, AIDS and STI Information System (OHASIS) as a Reporting Platform of HIV, AIDS, viral hepatitis and other STIs in the Philippines [11].
2. Joint DOH and PhilHealth Memorandum Circular No. 2021-0020. Mandatory Adoption and Use of National Health Data Standards for Interoperability [12].

Compared to other initiatives for creating data dictionaries for different disease entities, the following studies were identified:

1. A digital tool was developed using the WHO Antenatal Care (ANC) recommendations, which included identifying a minimum viable product, establishing antenatal workflows and algorithms, testing the established algorithms, developing a data dictionary and creating a front-end user interface together with application development [13].
2. The National Institute for Health Research Health Informatics Collaborative developed a comprehensive database that has been designed for potential viral hepatitis research interests. Here, they developed the data flow and data model for collecting viral hepatitis data [14].

### Scope and limitation(s) in the development of the data dictionary

It can be noted that the development of a data dictionary can vary from one study to another. The scope of this study will focus on the development of the data dictionary for resolving data fragmentation and standardization of terms being used through coding standards. In comparison with the digital ANC module, there was no mention of consensus building in their process, unlike in the methodology presented above. Moreover, case reporting and follow-ups were included in the data gathering to further encapsulate the context of the viral hepatitis initiative.

The study utilized only the following international standard code sets: the International Classification of Diseases 11th Revision (ICD-11), Logical Observation Identifiers Names and Codes (LOINC) and Systematized Nomenclature of Medicine Clinical Terms (SNOMED CT). Preference in using ICD-11 or LOINC took precedence over SNOMED CT because the Philippines is not yet a member of SNOMED International as of writing.

### Formulation of health data standards

**Figure 1:**
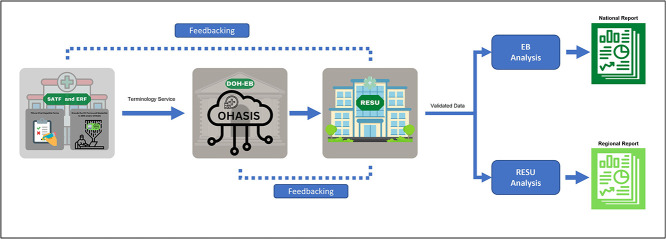
Viral hepatitis reporting flow adapted from the Overview of National Viral Hepatitis Surveillance [Google Slides presentation]. Department of Health, Philippines. Available from: https://docs.google.com/presentation/d/13aCS8CslZxxoBrhangXq0H0b_7COu5UEwS8QrqnGojk

Figure 1 shows the current reporting flow for viral hepatitis patients. Screening, assessment and treatment facilities and end-referral facilities accomplish the required forms by hand or using their own information systems, such as OHASIS [15]. Data from the information systems are then collected by the Epidemiology Bureau of the Philippine DOH for validation. The validated data are then forwarded to the Regional Epidemiology and Surveillance Unit for consolidation and further analysis.

The increase in the number of health information systems raises concerns about the interoperability of competing services. This prompted the DOH to issue policies, procedures and guidelines to eliminate duplication of information systems [11]. The Philippines Health Systems Review (under Health Information Management) published by WHO [16] and even the law entitled National Integrated Cancer Control Act (under Section 28) [17] have stipulated that all health-related facilities in the Philippines must adopt national health data standards.

**Figure 2:**
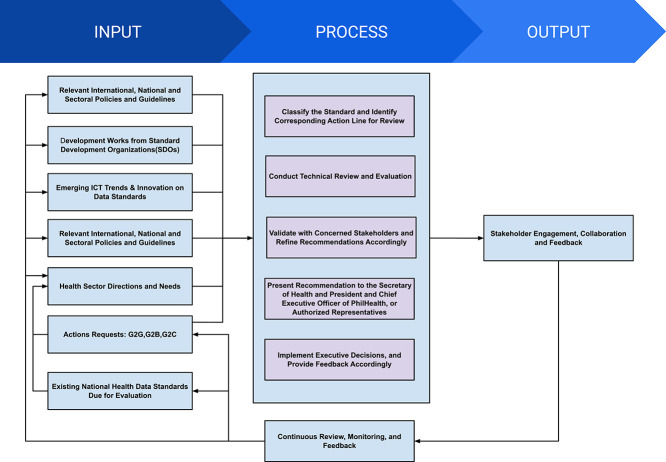
Implementing protocol on the adoption of health data standards adapted from Joint *Administrative Order No. 2021-0002 (Mandatory Adoption and Use of National Health Data Standards for Interoperability)* by DOH and PhilHealth, 2021. https://law.upd.edu.ph/wp-content/uploads/2021/06/DOH-PHIC-JAO-No-2021-002.pdf

Figure 2 details the implementing protocol of the DOH and PhilHealth [10]. In this figure, the ‘Input’ column illustrates the importance of stakeholders in updating and managing the data standards. On the other hand, the ‘Process’ instructs the National eHealth Program Management Office to conduct a technical review of the proposed changes. Finally, the ‘Output’ provides continuous feedback to ensure that the data standards are up to date.

## RESULTS

### Analysis of viral hepatitis forms for health data standards

As a notifiable disease, viral hepatitis cases are reported by healthcare providers to the Epidemiology Bureau of the Philippine DOH. Reporting is accomplished through the Viral Hepatitis Case Report and Care forms.

**Figure 3:**
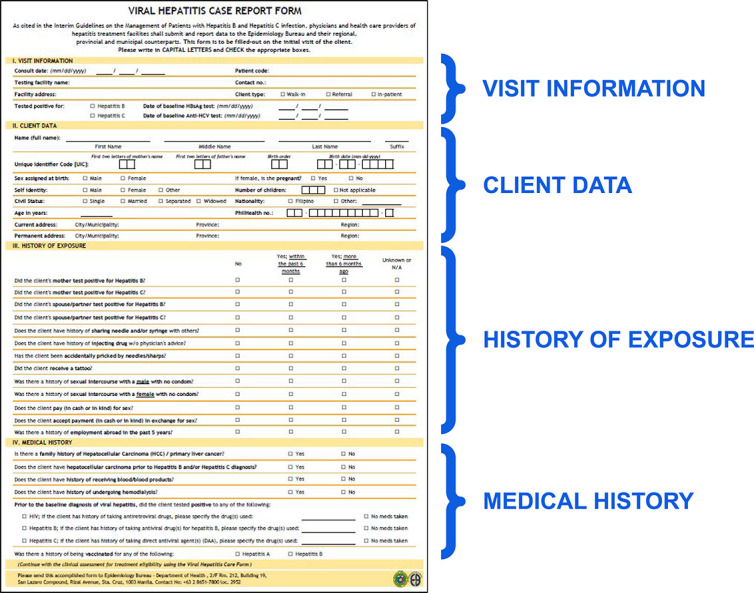
Hepatitis case report form adapted from the Overview of National Viral Hepatitis Surveillance. [Google Slides presentation]. Department of Health, Philippines. Available from: https://docs.google.com/presentation/d/13aCS8CslZxxoBrhangXq0H0b_7COu5UEwS8QrqnGojk

The Case Report Form (Fig. 3) collects several data, such as visit information, client data, history of exposure and medical history. Visit information provides context on the nature of the visit, such as the type and dates of viral hepatitis tests conducted. Meanwhile, client data collect patient demographic information. The history of exposure obtains information on close contact with hepatitis cases. Finally, medical history gathers relevant information that may influence the treatment plan.

**Figure 4:**
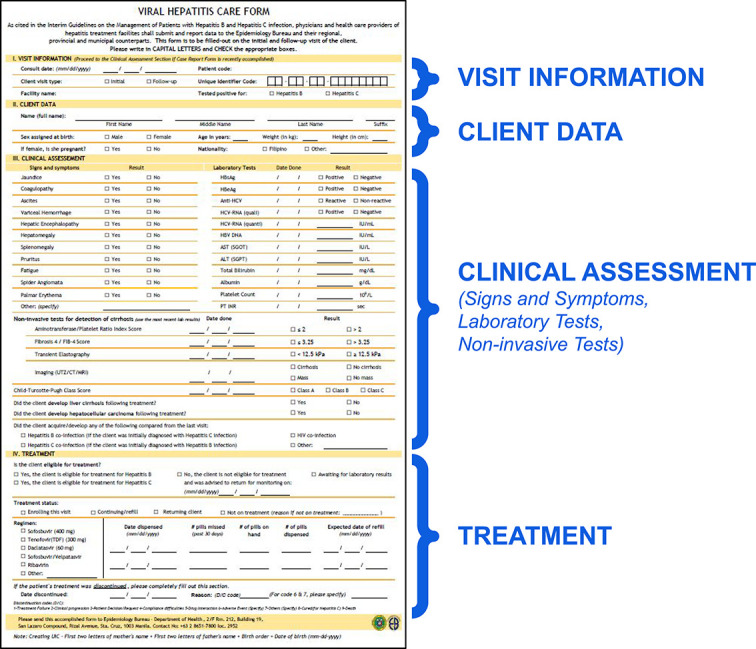
Hepatitis Care Form adapted from the Overview of National Viral Hepatitis Surveillance. [Google Slides presentation]. Department of Health, Philippines. Available from: https://docs.google.com/presentation/d/13aCS8CslZxxoBrhangXq0H0b_7COu5UEwS8QrqnGojk

The Care Form (Fig. 4) is accomplished by the healthcare provider to assess treatment eligibility at every patient visit. The form adds information on clinical assessment, listing the symptoms of the patient. The patient's treatment plan has been included as a section as well, specifying the dosage and frequency of medications.

Each data element identified for both the Viral Hepatitis Case Report Form and the Care Forms was eventually mapped to existing international standard code sets. During the selection of the code set, there was a preference for curated terminology over non-curated, international standards over local standards and free-to-use over pay-for-use terminology systems. Using these criteria, the selection process narrowed down the options to the following international standards—i.e. ICD-11, LOINC and SNOMED CT—as the main terminologies to be used. Health Level Seven-Fast Healthcare Interoperability Resources (HL7-FHIR) was selected as the standard for data exchange. These standards, their curators and their respective costs are shown in Table 1.

**Table 1:** Usage of selected international standard code sets

**Standard**	**Curator**	**Usage**	**Cost**
LOINC	Regenstrief Institute (loinc.org)	Laboratory observations	Free
ICD-11	WHO (icd.who.int)	Diagnosis of diseases	Free
Systematized Nomenclature of Medicine Global Patient Set (SNOMED GPS)	SNOMED International (snomed.org)	Terminologies	Free
SNOMED CT	SNOMED International (snomed.org)	Terminologies	By country membership
HL7 FHIR	HL7 International (hl7.org)	Data structure	Free or by membership

During the development of the proposed viral hepatitis data dictionary that will be reviewed for consensus of stakeholders, each data field that was present in either the Viral Hepatitis Case Report Form and Viral Hepatitis Care Form was assessed individually whether they existed on ICD-11 or LOINC—as curated, international and free code sets—and also through SNOMED CT—as a curated, international and paid code set while still staying true to the essence of the meaning of the term.

**Figure 5:**
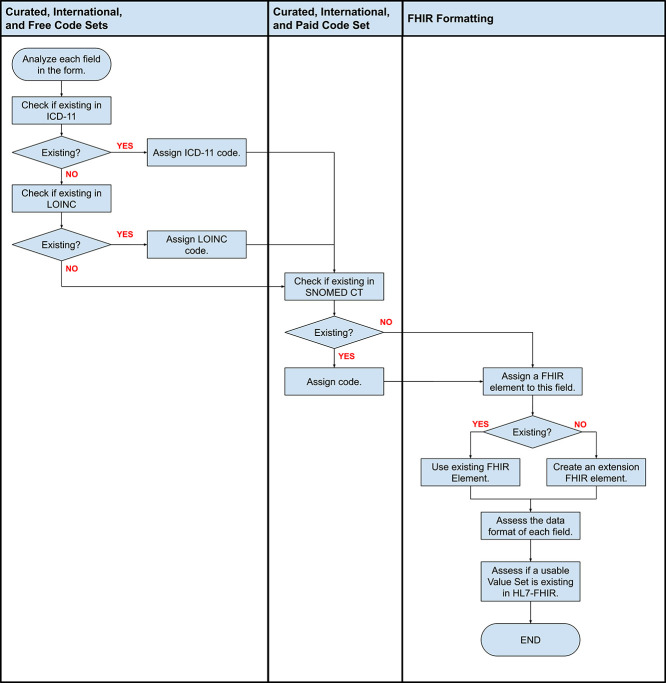
Criteria for selecting international code set for each data element

The algorithm in selecting the proposed international terminology code set in preparation for the consensus building is presented in Fig. 5. This algorithm demonstrates preference toward ICD-11 and LOINC since they are both free for use. As previously mentioned, during the preparation of the workshop, the Philippines is not yet a member country of SNOMED International, thus searching through either ICD-11 or LOINC took precedence over SNOMED CT. For cases wherein both free-for-use and pay-for-use international standard code sets are present, both codes and descriptions were presented to the stakeholders. Then, the Project Team provided an initial recommendation wherein the stakeholders can either agree or disagree to the recommendation.

The development of health standards requires a thorough review of the data collected in the hepatitis forms. A total of 125 unique fields in both forms were reviewed through a workshop with subject matter experts: epidemiologists and hepatologists. Specifically, the workshop aimed to select the appropriate international standard code sets presented to them, achieving consensus on the Philippine Viral Hepatitis Data Dictionary.

The initial workshop was conducted virtually with three breakout sessions represented by epidemiologists and hepatologists. During these sessions, the following steps were done:

1. Reiterate that the goal of the breakout session was to establish the codes for every data element assigned to the group.
2. Present each data element to the participants, showing both free-to-use and pay-for-use terminologies.
3. Present the recommendation from the Project Team with a preference for free-to-use over pay-for-use international terminology standard code sets.
4. Instruct the participants to accept or reject the recommendation. If they rejected it, they were asked to provide comments/suggestions on the data element.

**Figure 6:**
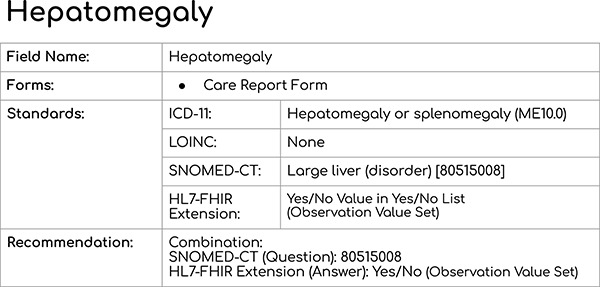
Sample data element presented during the breakout session

Figure 6 provides a sample presentation of how each data element was presented to the participants.

After the initial workshop, the majority of the viral hepatitis data elements achieved consensus (*n* = 87). Some data elements achieved consensus on the codes but had comments on the context of the data element itself (*n* = 15). On the other hand, some data elements required further discussion and were eventually parked (*n* = 17). A number of data elements were not reviewed at all and were parked (*n* = 6) because of time limitations at the workshop. Details of the results of the initial workshop are described in Table 2.

**Table 2:** Consultation results for the establishment of a viral hepatitis data dictionary

**Break out groups**	**Consensus**	**Minor comment**	**Suggestion**	**Not reviewed**	**Total**
**Group 1** *(Patient Demographics and Physical Examination)*	43	4	5	0	52
**Group 2** *(Medical History)*	8	0	11	6	25
**Group 3** *(Diagnostics and Therapeutics)*	36	11	1	0	48
**Total**	**87 *(69.60%)***	**15 *(12.00%)***	**17 *(13.60%)***	**6 *(4.80%)***	**125**

Upon consolidating the comments and suggestions of the participants from the different breakout groups, it was found that some of the suggestions stemmed from the content of the forms themselves and not from the proposed international terminology code sets. The consolidated feedback was then relayed to the DOH. Then, it was mutually agreed that there should be a reiteration that the consensus should focus on the proposed codes and not on the content of the form. Thus, a follow-up workshop was proposed, focusing on the international terminology code sets assigned to the forms instead of its content.

A follow-up workshop was held after 1 month where experts, via a consensus, resolved to modify several fields:

- Remove the units, in seconds, in ‘PT INR’ (Care Form)
- Remove: ‘Was there a history of employment abroad in the past 5 years?’ (Case Report Form) due to a lack of scientific basis
- Remove questions beyond the scope of the DOH Hepatitis program
  - ‘Is there a family history of Hepatocellular Carcinoma (HCC)/primary liver cancer?’ (Case Report Form)
  - ‘Does the client have HCC prior to hepatitis B and/or hepatitis C diagnosis?’ (Case Report Form)
- Revise names of fields with more appropriate terms
  - ‘Tested positive for hepatitis B’ (both forms) to ‘Tested positive for HBsAg test’
  - ‘Tested positive for hepatitis C’ (both forms) to ‘Tested positive for anti-HCV’
  - ‘Self-identity’ (Case Report Form) to ‘Gender Identity’
- Include ‘before 1995’ (prior to the regulation of blood banks [18]) to the question ‘Does the client have a history of receiving blood/blood products?’ (Case Report Form)
- Combine: ‘Was there a history of sexual intercourse with a male with no condom?’ (Case Report Form) and ‘Was there a history of sexual intercourse with a female with no condom?’ (Case Report Form) into a single question and add choices
- List the available treatment drugs in ‘If the client has history of taking antiretroviral drugs, please specify the drug(s) used:’ (Case Report Form)
- Rephrase questions that may single out certain groups and cause stigma
  - ‘Does the client have a history of injecting drugs without physician’s advice?’ (Case Report Form)
  - ‘Did the client receive a tattoo?’ (Case Report Form)
  - ‘Does the client pay (in cash or in-kind) for sex?’ (Case Report Form)
  - ‘Does the client accept payment (in cash or in-kind) in exchange for sex?’ (Case Report Form)
- Consider ‘Reactive/Non-reactive’ as choices on ‘HBsAg’ (Care Form) and ‘HCV-RNA (quali)’ (Care Form) aside from ‘Positive/Negative’
- Consider other dosages in ‘Regimen - Tenofovir (300 mg)’ (Care Form)
- Consider linking the patient to the Substance Abuse Treatment Facility or Rural Health Unit instead of ‘Current Address—City/Municipality’ (Case Report Form) and ‘Permanent Address—City/Municipality’ (Case Report Form)

The workshop displayed differences in priorities among subject matter experts. Epidemiologists are more concerned with obtaining as much data as possible for easier profiling of patients. On the other hand, hepatologists are more focused on protecting the personal information of patients. Nevertheless, reaching a consensus in all fields shows good promise for similar programs.

At the end of these workshops, all viral hepatitis data elements were assigned to an HL7 FHIR resource. However, not all data elements were assigned to an international terminology standard, mainly because of limitations identified in expressing the particular context of the data element. Details of the distribution of the mapping to the standards are listed in Table 3. For those data elements without international terminology code sets, they were still assigned a HL7 FHIR element to provide more context to the data element (e.g. Consult Date under Encounter resource).

**Table 3:** Number of Philippine viral hepatitis data elements mapped to an international standard and confirmed by experts

**International standards**	**Number of viral hepatitis data elements mapped to the standard (*N* = 125)**	
	***n***	**%**
HL7 FHIR *(Syntactic Standard)*	125	100
SNOMED CT *(Terminology Standard)*	60	48.00
LOINC *(Terminology Standard)*	21	16.80
ICD-11 *(Terminology Standard)*	19	15.20
No international standards terminology code set applied	25	20.00

## DISCUSSION

### Moving forward to computer processable clinical information

#### Translating the data dictionary into FHIR compliant requirements

While the Philippine Viral Hepatitis Data Dictionary has achieved consensus, planning for an implementation guide that will expose the data dictionary into a machine-readable format was prepared. The selected format for health information exchange is the HL7-FHIR Implementation Guide. This guide is a specification documentation on how FHIR resources (i.e. data formats) are used (or should be used) for a particular use case. It can be used by various stakeholders so they can embed the Philippine Viral Hepatitis Data Dictionary into their electronic data processing systems. A FHIR profile is organized and conforms to an existing FHIR resource, which, in turn, is a standardized data element format that is reproducible and expandable depending on the particular use.

**Figure 7:**
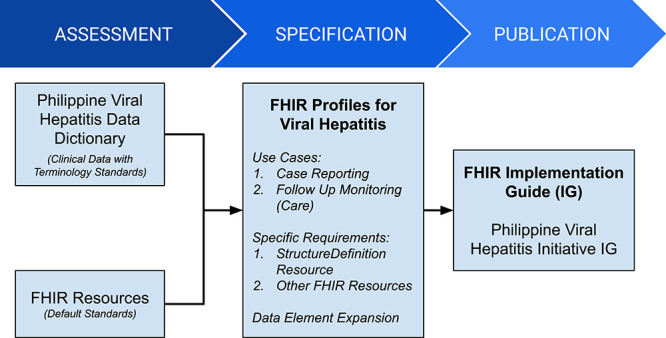
HL7-FHIR implementation guide development flow

In other words, whenever there is a need for interoperability, the process generally begins with understanding the decisions that stakeholders need to make. From these, the appropriate data set and terms can be identified and later mapped to reference terminologies. Once mapping to reference terminologies has been completed, these specifications should be translated to computable methods in order for these terms to be embedded into data processing systems. A simple diagram has been created to further encapsulate this process (Fig. 7).

#### HL7-FHIR manifesto

One of the core principles of HL7-FHIR is the 80/20 rule, wherein the focus of the interoperability standards is on the 20% of the requirements meeting the 80% of the interoperability needs. Because of this 80/20 rule, the FHIR resources were designed to meet the general requirements that cover many use cases to minimize the abundance, overlapping and redundancy of resources. Meanwhile, the concept of extension and customization has been implemented to utilize these general resources for more specific needs as required.

#### Connecting with international HL7-FHIR experts

Since implementing the data dictionary in the Philippines is not common practice nor has been institutionalized, a third-party company specializing in implementing FHIR was contracted to help in developing this data dictionary into computer-processable information that is accessible via health information exchange. Some of the high-level requirements were as follows.

1. Ability for participating clinics to access hepatitis B and C ‘Case Report’ forms online.
2. Ability for participating clinics to access hepatitis B and C ‘Care’ forms online.
3. Ability for participating clinics to write data to the demonstration FHIR server.
4. Ability for project owners to extract FHIR data from the demonstration server in a format suitable for data visualization or statistical computing tools like ‘R’ for statistical analysis.
5. Ability of participating clinics to read their data, including patient demographics, from the demonstration FHIR server.
6. Ability to limit the access to authenticated users only.

The six above-mentioned high-level requirements were established as part of the team’s requirements-gathering activities for the workflow of submitting cases to the DOH. The contracted expert third-party company provided a similar opinion. The progress of developing this solution for viral hepatitis is currently ongoing, particularly in establishing an FHIR server as well as concurring these mapped data elements with the third-party company into a machine-readable format.

## CONCLUSION

Semantic interoperability is important when consolidating non-standardized hepatitis data from different EMRs. To reach this state, prerequisite steps are needed. Identifying and convening the right stakeholders should be done first and is then followed by facilitating consensus on relevant data to monitor in the care of these patients. Once consenus has been achieved, the agreed-upon data sets and its associated international terminology code sets should be translated into standardized machine-readable formats. HL7 FHIR and other international systems such as ICD10, ICD11, LOINC and SNOMED offer standardized frameworks for creating machine-readable clinical concepts. Consensus of all stakeholders is crucial at every step of the process.

## Data Availability

The data underlying this article are available in the article.
